# First and Subsequent Lifetime Alcoholism and Mental Disorders in Suicide Victims With Reference to a Community Sample—the Lundby Study 1947–1997

**DOI:** 10.3389/fpsyt.2018.00173

**Published:** 2018-05-03

**Authors:** Cecilia Holmstrand, Mats Bogren, Cecilia Mattisson, Louise Brådvik

**Affiliations:** Division of Psychiatry, Department of Clinical Sciences, Skåne University Hospital, University of Lund, Lund, Sweden

**Keywords:** suicide, alcoholism, mental disorders, epidemiology, long-term course

## Abstract

**Background:** Suicide victims have been found to frequently suffer from mental disorders, often more than one, and comorbidity has also been found to be a risk factor for suicide. The aim of the present study was to determine the first disorder and possible subsequent disorders in suicide victims during their lifetimes and to compare their development with the development of mental and alcohol use disorders (AUDs) in a community sample.

**Methods:** The Lundby Study is a prospective longitudinal study of mental health in a general population comprising 3,563 subjects, including 68 suicide victims, followed by four field investigations from 1947 to 1997; mortality was monitored up to 2011.

**Results:** AUD was most common as a first diagnosis (26/68, 38.2%) among suicide victims, followed by “depression” (20/68, 29.4%) and “anxiety” (7/68, 10.3%). A predominance of AUD as a first diagnosis was found in the male group, whereas “depression” was the most common first diagnosis in the female group. However, there were very few females with AUD in the Lundby Study. In the whole population, it was more common for someone who started with an AUD to develop a subsequent mental disorder than the other way around. The same was true for AUD in relation to depression.

**Conclusions:** AUD was the most common first mental disorder among male suicide victims and could thus be considered a starting point in the suicidal process. We propose that in addition to detecting and treating depression, it is important to detect and treat AUD vigorously and to be alert for subsequent symptoms of depressive and other mental disorders in suicide prevention efforts.

## Introduction

Psychological autopsies have found mental disorders in approximately 90% of all suicide victims (1–6). In one review, only 0.1% of suicides in a psychiatric inpatient population and 3.2% of those in the general population had no established psychiatric diagnosis (7). Depression has often been reported as the predominant disorder, followed by substance use disorders (1, 5, 7–9).

Comorbidity of mental disorders is common among suicide victims (3, 5, 6, 10) and the most frequently reported combination is depression and alcohol/substance use disorder (6–9, 11). In a review of suicide risk in persons with depression, suicide was significantly more common in the presence of current substance misuse (12). Dumais et al. found that 41.3% of 104 male suicide completers who died during an episode of major depression had a life-time alcohol abuse/dependence (13). A long-term follow-up of depressives (38 years) showed an increased risk of suicide attempt among those who had developed a substance abuse (14).

In post-mortem case-control studies, comorbidity has been found more frequently in individuals who died from suicide than in controls. Comorbid depression is more common in subjects with alcohol/substance use disorder who have killed themselves than in controls (3, 8, 15) and alcohol abuse is found increased among first Major depressive episode suicide completers compared to controls (16). It has been suggested that an additive or synergistic effect of two disorders may enhance suicidality (17, 18). Comorbidity in the sense of co-occurring or overlapping mental disorders is most commonly described in the literature.

In one case-control psychological autopsy study, the author found that major depression was an important background factor in 41% of young suicide victims and secondary to other disorders in 19% of the victims. Alcohol dependence or abuse was found in almost 29% of the individuals and was mainly secondary (9).

Little is known about which disorder is the starting point of mental problems that eventually lead to suicide in the long-term perspective of adult life. The predominance of a mental disorder among suicides reflects its prevalence in the general population as well as the suicide risk for people with that disorder. For instance, depression, alcoholism, and schizophrenia have similar risks for suicide in the long-term course (19) but due to higher prevalence in the community depression and alcoholism predominate over schizophrenia among suicide victims (1, 7). The same holds true for comorbidity. If a certain temporal order of onset of mental disorders is found to predominate in suicides, this may reflect either a high risk for people with the first disorder to develop other disorders and thus a high prevalence of this order among comorbid cases. On the other hand, there may be a high risk for this order of comorbidity to end in suicide, regardless of the prevalence of this order. For instance, Alcohol use disorder (AUD) may more often be followed by depression than vice versa, or AUD followed by depression may be associated with a higher suicide risk than depression followed by AUD, both of which would be reason for a high incidence of a high rate of AUD followed by depression among suicide victims.

Comorbidity of AUD and depression, as well as possible links between these disorders, has been a focus of attention (20–25). In a 26-year-long Danish prospective cohort study, the authors reported that AUD was more likely to develop into another mental disorder than the other way around (26).

In summary, comorbidity of AUD and depression seem to be common among suicide victims. However, to the best of our knowledge, the long-term order of onset of AUD and other mental disorders has not been studied in suicides. Moreover, no one has studied whether AUD or another mental disorder comes first in relation to suicide risk among comorbid cases. Knowledge of the lifetime history of mental disorders and of the first episode could be important in secondary prevention of suicide (27–29).

The Lundby Study is a long-term prospective community study that began in 1947, with follow-ups in 1957, 1972, and 1997. Mortality was monitored up until 2011, a 64-year follow-up. Thus, we could detect a first diagnosis even a few decades before suicide. Previous reports from the Lundby Study have shown an elevated suicide risk in individuals with only depression (6.0%), only AUDs (4.7%), only psychosis (3.1%) (30) and only anxiety (2.5%) (31). Additional diagnoses increased the risk significantly, and there was a very high risk of suicide (16.2%) among men with AUDs and depression (30), order of onset not considered. In another study of the Lundby population, depression predominated among suicide victims, followed by AUD. Comorbidity was more common in the alcohol group, but long-term course and temporal order of diagnoses were not considered (32). The Lundby Study provides an opportunity to examine the development of psychiatric disorders in a general population with reference to suicide.

The aims of the present study were to find out about the life-time history of mental disorders and the first episode and subsequent comorbidity in suicide victims and in the general population. First, the first lifetime diagnosis and subsequent diagnoses in suicide victims were investigated. Second, the temporal order of AUD and other mental disorders was investigated (a) among suicides, (b) among persons with AUD and any other disorder, as an impact of suicide risk, and (c) in the community population, as on impact on prevalence. AUD in relation to depression was investigated separately.

## Materials and methods

### The lundby population

The prospective Lundby Study was started in 1947 by Essen-Möller and his colleagues with the idea of describing the mental health and personality traits in a general population in a geographically defined rural area near Lund, in the southern part of Sweden (33). The investigators studied all inhabitants, regardless of age. Field investigations were based on interviews by psychiatrists (a semi-structured part and a free part), in which the subjects were asked about their lives and health situation.

**Figure 1:**
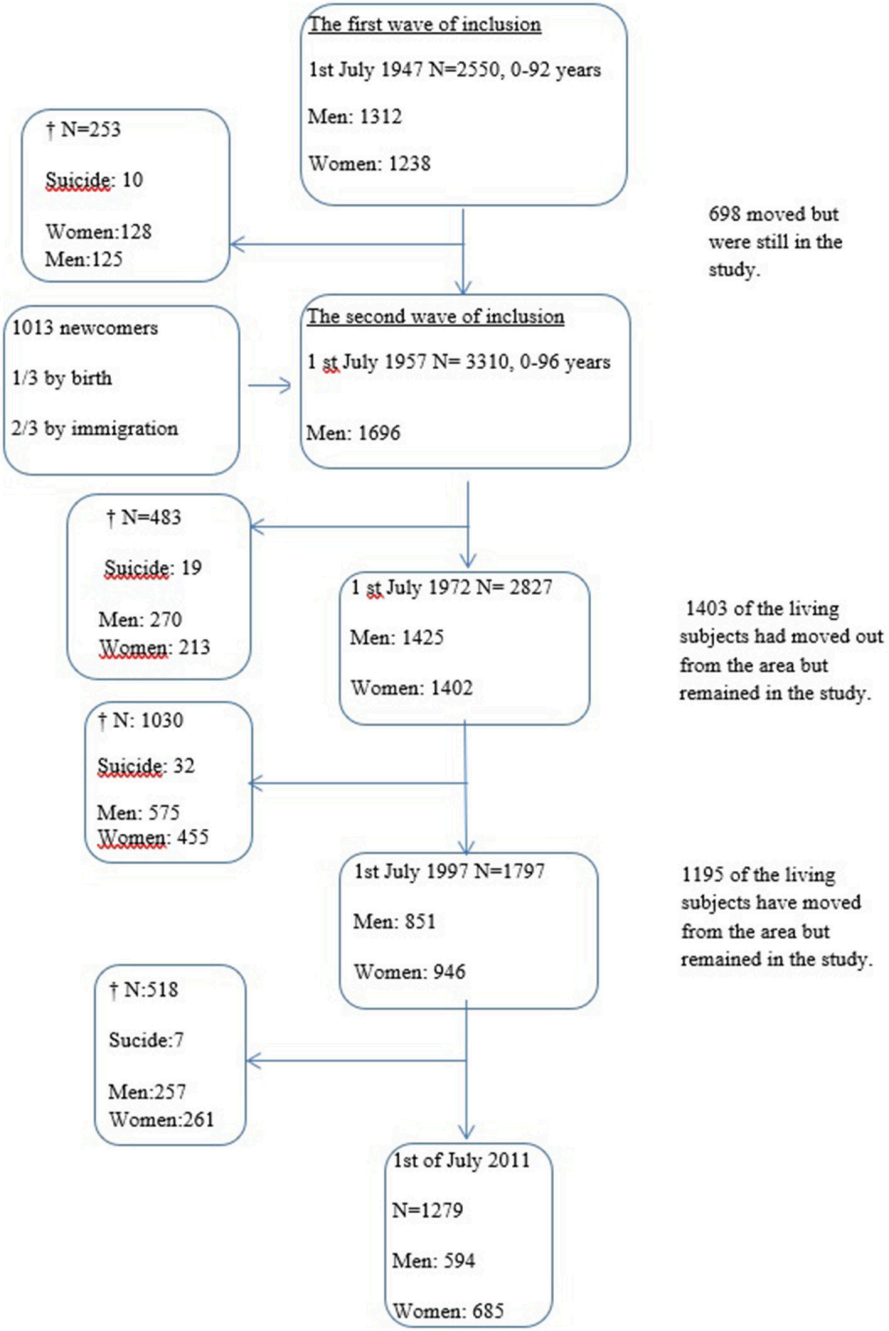
The Lundby population—inclusion, deaths and attrition during the follow-up 1947–1997.

The original sample consisted of 2,550 people. In a follow-up carried out in 1957, 1013 new inhabitants, who had either moved into the area or been born there after 1947, entered the study, making two overlapping cohorts with a total of 3,563 individuals (Figure 1). Surviving subjects were asked to participate again in 1957, 1972, and 1997. Field investigators traveled to visit those who had moved from the area or, if that was not possible, conducted the interview by telephone. The interviews were supplemented with information from key informants (for example, family members, clerics, local physicians, teachers, foremen) and outside sources, such as diagnoses from the National Patient Register, a local in-patient register, the County Temperance Board (until 1983) and the Cause of Death Register.

During the follow-up period to 1997, 1766 individuals died.

In 1997, the drop-out rate (refusal or insufficient information about the subject) was 8.7% among living subjects and 5.9% when both living and dead subjects were included, but these figures were still low according to common standards (34).

In the latest field investigation, in 1997, the area had developed from rural to mainly suburban.

### Diagnostic procedure

In 1947 and 1957, there was no established diagnostic system suitable for a field study of a general population. Professor Essen-Möller devised his own “Lundby diagnostic system,” which was further developed in 1957 together with co-investigator Hagnell. This system included disorders in a primary group called “Diagnosis I,” which was meant to represent acquired disorders with a perceptible onset and mostly termination. This is a hierarchical system allowing only one diagnosis per episode; for example, dementia rules out psychosis. Habitual conditions rooted in personality, alcoholism and psychosomatic complaints were consolidated in the “Diagnosis II” group. In the Lundby classification, a subject could be given one Diagnosis I for an episode of a mental disorder and one or more Diagnosis II during the same period of time.

In this study, the main Lundby Diagnosis I, and AUD from Diagnosis II, are used. In the 1997 field investigation, diagnoses according to Lundby, the DSM IV (35) and the ICD-10 (36) were assessed for the period 1972-1997. Re-evaluations of several Lundby diagnoses from earlier field investigations have also been made (37). In the present study, we chose to use the Lundby diagnoses, as not all episodes of mental disorders have been re-evaluated.

### Degree of impairment

The functional impairments of mental disorder episodes were assessed according to Leighton et al., who defined five levels of impairment (38). Medium impairment (level 3) or higher has previously been used as a criterion for mental illness or “caseness” in subjects with a Lundby diagnosis (37, 39). This corresponds to a GAF score between 60 and 51 or less (35).

### Lundby diagnoses

“*Alcohol problems” (in the present paper defined as AUD)* is defined as a persistent pattern of recurrent alcohol use resulting in failure to fulfill major obligations at work, school, or home; i.e., the subject may use alcohol even in a physically hazardous situation (such as driving a car), there may be recurrent alcohol-related legal problems (such as arrest for alcohol-related disorderly conduct), and the alcohol use is persistent despite social and interpersonal problems caused by the effects of alcohol use.

“*Alcohol dependence” (in the present paper defined as AUD)* is defined by DSM-IV criteria as a maladaptive pattern of alcohol use leading to clinically significant impairment or distress, as manifested by at least three of seven criteria occurring at any time during a 12-month period.

“Alcohol problems” in the Lundby Study are similar to the DSM-IV criteria for alcohol abuse. In this study, we use the broader category “alcohol use disorder” or AUD, which is used if a participant met the criteria for “alcohol problems” or “alcohol dependence” at any time between 1947 and 1997. In addition, an estimated duration of disorder of at least one year was a criterion.

In 28 of the subjects with AUD onset before inclusion in the study, the age of onset is unknown. In these cases, the age of onset is regarded as the age at inclusion.

There were 36 persons (19 women and 17 men), who had a problem use of prescribed drugs in 1997.

Seven of them committed suicide and five used alcohol as well. Three of the persons with AUD had comorbid diagnoses, in two of them the other diagnosis preceded the AUD. No suicide cases had used illegal drugs up to 1997. Two non-suicides used amphetamine.

“*Depression”* is defined by the Lundby diagnosis as follows:

*Lowered mood, depressive feelings, tendency to feel guilt, gloomy outlook, reduced activity, lack of initiative, reduced self-esteem, lowered enjoyment of life and a feeling of low vitality, anxiety and fear. Has more difficulty than usual and is often unable to carry out his daily responsibilities. Sometimes retardation is present. The subject is often worse in the morning and better toward the evening. Often he has sleep disturbances and wakes up in the early morning. Loss of appetite and weight (40)*.

Subjects with “depression with psychotic symptoms” were included in the “depression” group, according to the DSM-IV main-classification of “mood.” In the majority of cases, the depression group could be approximated to major depression in DSM-IV as a medium and severe degree of impairment (see above), but sometimes it corresponds better to dysthymia, depression NOS (Not Otherwise Specified). Individuals with bipolar disorder (12 individuals) are also included.

“*Organic brain disorder”* represents a class of disorders with clearly disturbed cognitive functions or a clear deterioration of personality or behavior in which the etiology is unquestionably organic. The sub-category “dementia” in the Lundby Study comprises Alzheimer's disease, multi-infarct dementia and other varieties of dementia (40).

*Psychosis* in the present study include schizophrenia and other psychoses (41). “Anxiety” corresponds with panic disorder, generalized anxiety disorder, phobias, etc. (42).

“*Other psychiatric disorders”* in this study represents “Tiredness” and “mixed neurosis” The group is heterogeneous and include diagnoses that, according to DSM-IV, could be classified as social phobia and somatoform/somatization disorder, adjustment disorders, phobias, sleep disorders or anorexia nervosa (40, 42–44).

At the time of the latest check of the National Cause of Death Register (45) on 1 July 2011, 2284 of the 3563 individuals had died (64%, Figure 1).

### Criteria for suicide

In the Lundby Study, we have followed the often-used approach to include cases of death by undetermined intent in the suicide group, as similarities have been found between the two groups in several studies (46–48). On the other hand, differences in background variables between deaths classified as suicide and deaths classified as “undetermined intent” have been highlighted recently (49). The similarities between accidental overdoses and death by undetermined intent rather than by suicide in substance use disorder have also been noted (50). We therefore considered suicide separately.

Suicide was defined by the International Classification of Diseases (ICD) codes, from revisions 6–10 (36, 51–54). Information about suicides was retrieved from the Cause of Death Register (45). Until 1994, classification codes E950-E959 represented “suicide and self-inflicted injury,” and E980-989 represented “injury undetermined whether accidentally or purposely inflicted” in ICD 8 and ICD-9. From 1994 to 2011, ICD 10 was used, and the categories X60-X84 represented “intentional self-harm,” and Y10-Y34 represented “events of undetermined intent.”

By 1 July 2011, a total of 68 suicides had occurred (51 men and 17 women), including 19 deaths by undetermined intent.

### Statistical methods

We used Fisher's exact test when examining the category variables. Statistical significance was set for *p*-values below 0.05.

### Ethical statement

The Research Ethics Committee of the Medical Faculty at the University of Lund approved the 1997 follow-up of the Lundby Study, and the participants provided written consent.

## Results

### First lifetime diagnosis and subsequent diagnoses among suicide victims

Sixty-two of the 68 suicide victims (91.2%) had one or more diagnoses. Thirty-nine individuals had only one diagnosis (57.3%), but subsequent diagnoses during the observation period were found in 23 individuals (33.8%), in 21 of the 51 (41.2%) male suicides and in two of the 17 (11.8%) female suicides.

**Figure 2:**
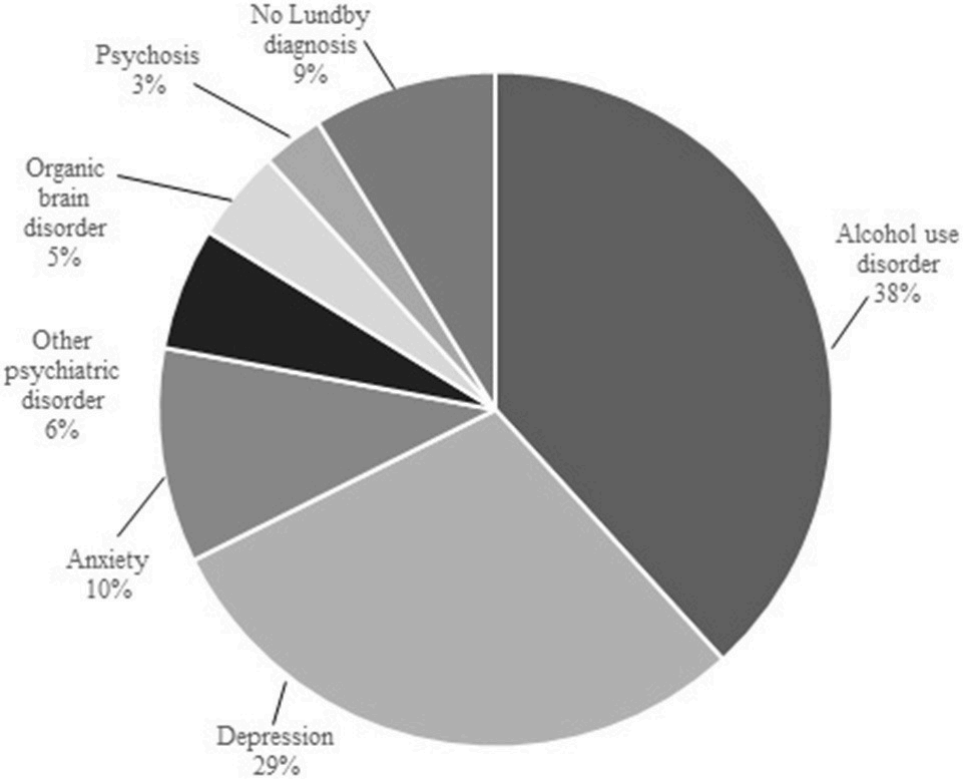
The distribution of first diagnoses in suicide victims.

AUD was most common as first diagnosis (26/68, 38.2%), followed by “depression” (20/68, 29.4%) and “anxiety” (7/68, 10.3%). The distribution of onset diagnoses in the suicides is presented in Figure 2. When studying males and females separately, we found that the dominance of AUD first in the observation period was only found in male suicide victims. “Depression” was the most common first diagnosis among 17 female suicide cases, followed by “anxiety.” The first and subsequent diagnoses during the lifespan among suicide victims are presented in Table 1.

**Table 1:** First and subsequent diagnoses of suicide victims (*n* = 68).

**First diagnosis (*n* = 68)**	**Subsequent diagnosis I (*n* = 23)**	**Subsequent diagnosis II (*n* = 3)**	**Subsequent diagnosis III (*n* = 1)**	**Subsequent diagnosis IV (*n* = 1)**
Organic brain disorder, 3	Depression, 1			
Psychosis, 2				
Depression, 20	Organic brain disorder, 1			
	Alcohol use disorder, 2			
Anxiety, 7	Psychosis, 1	Depression, 1		
	Depression, 1			
	Alcohol use disorder, 1			
Alcohol use disorder, 26	Depression, 7			
	Psychosis, 6	Depression, 1	Anxiety, 1	Other psychiatric disorders, 1
	Organic brain disorder, 1	Depression, 1		
	Anxiety, 1			
Other psychiatric disorders, 4	Anxiety, 1			
No Lundby diagnosis, 6				

Comorbidity between AUD and “depression” was most common and was present in 11 (16.2%) cases.

When studying the male suicide victims (51), excluding cases of undetermined intent, AUD was the most common first diagnosis (15/31, 48.4%), followed by depression (11/31, 35.5%).

### Temporal order of comorbid disorders in persons with AUD

#### Suicide victims

Among the suicide victims, the most common situation was that persons began with AUD and then developed a mental disorder. It was less common to start with another mental disorder and subsequently develop AUD (15/26, 57.7% vs. 3/36, 8.3%, Fisher's Exact, *p* < 0.001). None of the individuals with AUD first had a remission between the AUD and the start of the subsequent diagnosis, and only one of the persons with AUD coming after another mental disorder had a remission of their first diagnosis before developing AUD.

We found a non-significant trend indicating that it was more common to have AUD first and subsequently develop “depression” (9/26, 34.6%) than the other way around (2/20, 10.0%, Fisher's Exact, *p* = 0.08). One of the two persons with “depression” before developing AUD had a remission between episodes.

Among suicide victims, there was only one woman with AUD (no long-term comorbid diagnosis), so it was not possible to compare genders.

There was no significant difference in the occurrence of additional diagnoses during the observation period among persons with AUD in the group of undetermined intent (8/13) compared with the suicide group (10/16, Fisher's Exact, *p* = 1), and there was no significant difference when AUD was first in those long-term comorbid persons between the undetermined intent (6/8) and the suicide group (9/10), Fisher's Exact, *p* = 0.559).

#### Subsequent diagnoses and suicide

When studying the 191 individuals with AUD and long-term comorbid mental disorders, no significant difference was found between the proportion of individuals who had killed themselves in the group who had started with AUD (15/127, 11.8%) and in those who had started with another mental disorder (3/64, 4.7%, Fisher's Exact, *p* = 0.125).

In persons with both AUD and depression (*N* = 85 persons) during the follow-up, nine of the 54 persons (16.7%) with AUD first had killed themselves, whereas two of the 31 persons (6.5%) with AUD following “depression” committed suicide. However, the proportions of suicide did not differ significantly between the groups (Fisher's Exact, *p* = 0.314).

#### Subsequent diagnoses in the general population

In the total Lundby population, it was more common for a person who started with AUD to develop a subsequent mental disorder (127/363, 35.0%) than for a person with a mental disorder to subsequently develop AUD (64/1155, 5.5%; Fisher's Exact, *p* < 0.001).

There was also a higher proportion of individuals who had an AUD and subsequently developed “depression,” 54/363 (14.9%), than the other way around, 31/384 (8.1%; Fisher's Exact, *p* = 0.004). None of those with AUD as a first diagnosis had remission before the onset of depression. Seven persons had a remission of depression before developing AUD.

## Discussion

Mental disorders have been found among a clear majority of suicide victims, and comorbidity is common, especially between depression and AUD. Comorbid mental disorders are also associated with an increased risk of suicide in the long-term course. However, we believe there has been a gap in the knowledge concerning the development of comorbidity stating which mental disorder precedes the other as a starting point in the suicidal process.

First, among suicide victims, AUD was the most common first diagnosis and depression second-most. Most suicides had no subsequent diagnoses, but AUD with subsequent depression was the most common comorbidity. Moreover, among suicides with a subsequent diagnosis, AUD was usually the first diagnosis.

Second, we could not conclude whether AUD first or later in the mental disorder history (depression or any disorder) of those who had two disorders should imply a higher suicide risk.

Third, in the whole Lundby population, AUD was more likely to precede another mental disorder than the other way around. Specifically, AUD was more common prior to depression rather than after it. Most episodes temporally overlapped, especially when AUD had preceded another mental disorder.

Thus, the finding that AUD usually precedes other disorders seems to reflect a higher prevalence of this order rather than higher suicide risk for this temporal development of the disorders.

Previous studies on mental disorders in suicide victims have focused mainly on diagnoses at the time of death without considering the long-term development of mental disorders before suicide. Depression has been reported as the most common diagnosis (35.8 to 87%) among suicide victims, followed by substance use disorders, in both clinical and general populations (1, 5, 7, 8) and in young people (9). However, in the present study, AUD was the most common first diagnosis in in male suicide victims, and depression was the most common first diagnosis in female suicide victims. Conclusions could not be drawn for women due to the small sample size, but gender differences regarding mental disorders in suicide cases are in accordance with a previous meta-analysis (1).

Psychiatric comorbidity is common among suicide victims, but the order of the comorbid conditions seems to be less frequently studied. In a Taiwanese psychological autopsy study, Cheng found that most suicide victims with AUD had comorbid disorders and that their clinical history usually indicated that the onset of alcoholism AUD preceded the depression (8). The latter finding accords with the present study. In a very long-term study (48–52 years after hospitalization for depression) by Angst et al. the increased risk of suicide was higher in major depressive disorder (SMR 23.1) than bipolar I disorder (SMR 12.0) (55). In a previous study by the same group suicide attempt was found to be related to depression and anxiety as well as substance use disorders (14).

We have found few studies on the risk of suicide among patients with comorbid diagnoses and risk of suicide related to temporal order, and we could not conclude any impact of the order of AUD and other diagnoses from the present findings. However, the small sample size do not allow us to exclude an increased risk of suicide among persons with AUD, who develop a depressive disorders. The Lesch typology of alcohol dependence describes a stage III with major depression, a phase where alcohol is used as self-treatment. This phase is also associated with severe suicidal ideas in the absence of alcohol (56, 57). This model may explain the increased risk of suicide among people developing depression secondary to AUD.

In this study, we found that many individuals in the Lundby population with both AUD and one other mental disorder had AUD as a first diagnosis. Similar results have also been presented in a large prospective Danish study (26) but we have examined mental disorders in a general population in a very long-term perspective study. On the other hand, The National Comorbidity Survey concluded that AUDs are usually temporally secondary (58).

We also found that it was more common in the total Lundby population to have had AUD first and subsequently suffer from depression, compared to having AUD after an episode of depression. These results are consistent with some other studies. A study based on the National Epidemiological Survey on Alcohol and Related Conditions (NESARC) showed that alcohol abuse, but not alcohol dependence, preceded many mood and anxiety disorders, with lag times between 7 and 16 years (59). Further studies have highlighted that high alcohol consumption/AUD increases the risk of depression (60, 61).

In contrast, other studies indicate that depression prior to AUD is more common than the other way around (62, 63) and may be more serious because of suicidal ideas or behavior (18, 64). In addition, depression predicts future alcohol use and impairment (23).

In the Epidemiologic Catchment Area Survey in the US, the authors found that both alcohol dependence and major depression pose a significant risk for the development of the other disorder at 1 year, but more so in women (65).

The bidirectional relationship between alcohol dependence and major depression was examined in a study of a general population in which alcohol dependence increased the risk of major depression, and major depression increased the risk of alcohol dependence in men (66).

Some controlled clinical trials have shown that antidepressant treatment will reduce depressive symptoms in person with co-occurring depression and alcohol dependence (67) and untreated depression predicted worse drinking outcomes (68). Suicidality in alcohol-dependent individuals has been reviewed, and the conclusions were that suicidal communication should be taken seriously, other mental disorders should be carefully evaluated, and both conditions should be treated (69). Our results show that AUD often preceded depression in persons who later killed themselves. Thus, we agree with this conclusion and want to stress the importance of observing individuals with AUD and subsequent mental disorders for further loading factors in secondary suicide prevention. More detailed studies on age of onset, sub-types of alcohol dependence, possible long-term interplay between AUD and other mental disorder or other substance use, as well as possible links to suicide would be important topics for future research in long-term studies, i.e., the Lundby study (Structural equation modeling (SEM) may be used).

### Strengths and limitations

The major strength of the present study is the very long-term follow-up, 64 years, approaching life-time. The major limitation is the small sample size of only 68 suicides. There were few cases of comorbid AUD and depression, which makes these analyses non-conclusive. Finally, there were few female suicides.

Prospective diagnostics minimize the risk of recall bias and bias of knowledge of the suicidal outcome, which is a problem in psychological autopsies. The attrition rate is low in the Lundby Study, which, together with the study of a complete population, minimizes selection bias.

Further strengths are that all evaluations were made by psychiatrists who carried out the interviews. The use of multiple sources to collect information in addition to the interviews is another strength, which reduces the risk of recall bias.

The interviews in this longitudinal study were carried out with relatively long intervals of 10, 15, and 25 years between them, which could be a limitation. A major limitation is the small sampling size of the suicide victims, especially for the female group.

Psychiatric illness among participants before entry into the study could have been missed. However, as AUD often began earlier than depression, such cases may not invalidate results. The Lundby Study began before structured instruments were widely applied, which is a limitation, and thus, a standardized validated interview was not used. DSM-IV diagnoses were only applied during the period from 1972 to 1997, and other DSM diagnoses were added in retrospect.

In this study we have not investigated possible subtypes of drinking pattern or origin of alcohol craving in as described in A and B subtypes of alcohol dependence (70) or subtypes according to Lesch in the subjects with “alcohol use disorder” (57). We might have identified untreated individuals or those difficult to treat with Lesch type III alcoholism among the suicide victims with long-term “alcohol use disorder” and “depression” comorbidity. As AUD started first in most of the suicide victims, the depression might have been undetected and untreated in these individuals.

There were few cases of abuse of legal and illegal drugs. These were only investigated at the fourth field-investigation in 1997 and it is not likely that illegal drugs confounded the results earlier in the study as they were very uncommon, maybe absent, in the population early in the study. However, some use of prescribed drugs may have been undetected in earlier follow-ups. In two of three suicide cases with comorbid AUD and other diagnoses, the other diagnosis preceded the AUD. It could be assumed though not be proven, that comorbid recreational use of legal drugs was not an important confounding factor in the present study. No suicide had used illegal drugs.

Finally, the possible influence of sociodemographic risk factors was not investigated in the present study. However marital and socioeconomic status have been investigated previously in the Lundby study and these factors did not differ significantly in suicide cases compared to others in the Lundby population (71).

## Conclusion

Suicide victims are known to have suffered from mental disorders before suicide. Depressive disorders are generally considered to be the most common, and detection and treatment of depression is important in suicide prevention efforts. In the present study, AUD was the most common first diagnosis in male suicide victims and could therefore often be seen a starting point in the suicidal process. Therefore, while depression is common, the significance of AUD should not be overlooked. Accordingly, the treatment of AUD and monitoring the development of subsequent mental disorders, especially depression, seem to be of major importance in suicide prevention at the secondary and tertiary levels.

## Author contributions

LB conceived the study and contributed to the analyses. CH contributed to the design and drafted the manuscript. CM and MB performed most interviews during the fourth field investigation and contributed to the analyses. All authors read and approved the paper.

### Conflict of interest statement

The authors declare that the research was conducted in the absence of any commercial or financial relationships that could be construed as a potential conflict of interest.
